# 150th anniversary of The Physiological Society. Building on the breakthroughs of Dr Florence Buchanan: Advancing the frontiers of muscle physiology

**DOI:** 10.1113/EP094458

**Published:** 2026-10-22

**Authors:** Damian M. Bailey, Colleen S. Deane, Jakob Škarabot, Grith Højfeldt, Guido Ferretti

**Affiliations:** ^1^ Neurovascular Research Laboratory Faculty of Life Sciences and Education University of South Wales Pontypridd UK; ^2^ Bexorg, Inc. New Haven Connecticut USA; ^3^ Human Development and Health, Faculty of Medicine University of Southampton, Southampton General Hospital Southampton UK; ^4^ School of Sport, Exercise and Health Sciences Loughborough University Loughborough UK; ^5^ Institute of Sports Medicine Copenhagen Department of Orthopaedic Surgery Copenhagen University Hospital–Bispebjerg and Frederiksberg Copenhagen Denmark; ^6^ Department of Clinical Medicine Faculty of Health and Medical Sciences University of Copenhagen Copenhagen Denmark; ^7^ Department of Molecular and Translational Medicine University of Brescia Brescia Italy

The Physiological Society was founded in 1876 to advance physiology and to facilitate exchange between physiologists. One hundred and fifty years later, that same purpose remains remarkably intact. The questions have evolved, the instruments have been transformed, and the physiological community now reaches far beyond anything its founders could have imagined. Yet the discipline still draws its strength from the same basic ingredients: measuring carefully, interpreting rigorously, sharing methods openly, and gathering in person to challenge, refine and advance ideas.

When the Physiological Society invited the organisation of a symposium for its 150th anniversary programme, Dr Florence Buchanan's work and legacy were the obvious starting point: few figures link the Society's history, the origins of *Experimental Physiology* and the continuing vitality of muscle physiology so directly. It was in this spirit that a special symposium, ‘Building on the breakthroughs of Dr Florence Buchanan: advancing the frontiers of muscle physiology’, was held on 9 September 2026 during the joint congress of the Federation of European Physiological Societies and the Italian Society of Physiology (FEPS–SIF) in Brescia, Italy. The setting could hardly have been more appropriate. Brescia provided an especially fitting setting. Its layered Roman, Venetian, industrial and scientific histories reflected the broader theme of the meeting: physiology as a discipline strengthened by exchange across borders, institutions and traditions. Bringing The Physiological Society's 150th anniversary to Brescia also reflected the Society's strong and growing relationship with Italian physiology, a relationship recently marked by the award of the first international Physiological Society Blue Plaque outside the UK and Ireland in honour of Professor Rodolfo Margaria (1901–1983) at the University of Milan (Minetti et al., 2026). In this respect, FEPS–SIF 2026 did more than simply provide a venue for the symposium; it framed Dr Buchanan's legacy within a broader European tradition of physiological exchange, collaboration and discovery.

That Italian tradition is exceptionally rich. Among the founders of modern physiology, during the resurgence of scientific inquiry from the 17th century onwards, stand Santorio Santorio (1561–1636), who first tried to measure heat production by contracting muscles; Giovanni Alfonso Borelli (1608–1679), who first analysed animal movement in terms of mechanical laws; and Marcello Malpighi (1628–1694), who first described the lung alveoli. This tradition continued through the pioneering work of Angelo Mosso (1846–1910) on muscle fatigue, circulation, respiration, exercise and high‐altitude physiology, and later through Rodolfo Margaria's Milanese School, together helping to define the scope of modern integrative human physiology (Bailey et al., 2026; Ferretti, 2023). The Milanese School – shaped by Margaria and carried forward by figures including Professors Emilio Agostoni (1929–2021), Giovanni Cavagna (1934–2025), Paolo Cerretelli (1932–2026), Franco Saibene (1932–2008) and Pietro E. di Prampero, who attended the Brescia meeting at the age of 86 – helped establish modern applied, exercise, locomotor and environmental physiology (Bailey, 2026; Ferretti, 2023). That legacy continues through contemporary Italian physiologists whose work has shaped muscle bioenergetics, oxygen transport, locomotion, ageing and space physiology, including Professors Carlo Capelli, Fabio Esposito, Guido Ferretti, Bruno Grassi, Marco Narici and Paola Zamparo, whose presence in Brescia provided a direct link between this distinguished scientific lineage and its continuing evolution. The symposium therefore connected Dr Buchanan's experimental legacy with Italy's long‐standing strength in integrative human physiology.

The symposium was anchored in the life and work of Dr Florence Buchanan (1867–1931), one of the most striking figures in the history of British physiology. Dr Buchanan was the first woman known to have attended a meeting of The Physiological Society, among the first of six women elected to membership in 1915, and the author of the paper that opened the first volume of *The Quarterly Journal of Experimental Physiology*, the direct predecessor of *Experimental Physiology* (Ashcroft, 2022; Bailey, 2023, 2026; Buchanan, 1908; Clark, 2025; Clark et al., 2026; Tansey, 2015). That 67‐page study, based on 57 experimental preparations, used the capillary electrometer to resolve an early direct motor response from a later spinal reflex response in the frog gastrocnemius and, after correcting for peripheral nerve conduction, provided one of the earliest quantitative estimates of the delay introduced by central spinal transmission (Buchanan, 1908).

The achievement was technical, but its significance was far broader. Dr Buchanan showed how a difficult physiological question could be made experimentally tractable through the right combination of preparation, instrumentation and interpretation. In Dr Buchanan's hands, fleeting electrical events in nerve and muscle were converted into durable physiological records. Her work sat firmly in the Carl Ludwig (1816–1895) tradition (Bailey, 2026), where traces made millisecond events available to measurement, comparison and interpretation. From marine invertebrate morphology to muscle bioelectricity, reflex transmission, cardiac rhythm, pulse frequency and exercise physiology, Dr Buchanan exemplified an integrative style of pioneering science: one that linked structure to function, instrumentation to insight, and experimental precision to biological meaning and clinical translation.

The opening contribution by Professor Damian M. Bailey, ‘From reflex arcs to missing faces: The science and legacy of Dr Florence Buchanan’, revisited this dual inheritance. On the one hand, Dr Buchanan helped establish the temporal logic of reflex physiology: peripheral conduction alone could not explain the observed delay, leaving central spinal processing as the essential physiological event (Buchanan, 1908). On the other hand, her career openly exposed the asymmetries of recognition that shaped early scientific life. She could contribute to the Society, but not fully belong to it; she could open a journal's published record, yet disappear from the visual record. Recent work sought to correct that physical absence, first through an AI‐assisted, evidence‐informed attempt to visualise her historical presence, and subsequently through the discovery of an authenticated portrait in a Buchanan family memoir (Bailey, 2026; Clark et al., 2026). Figure 1 does more than simply illustrate the past; it allows *Experimental Physiology* and The Physiological Society to recognise its first author not only through her published work, but as a fully visible figure in its history.

**FIGURE 1:**
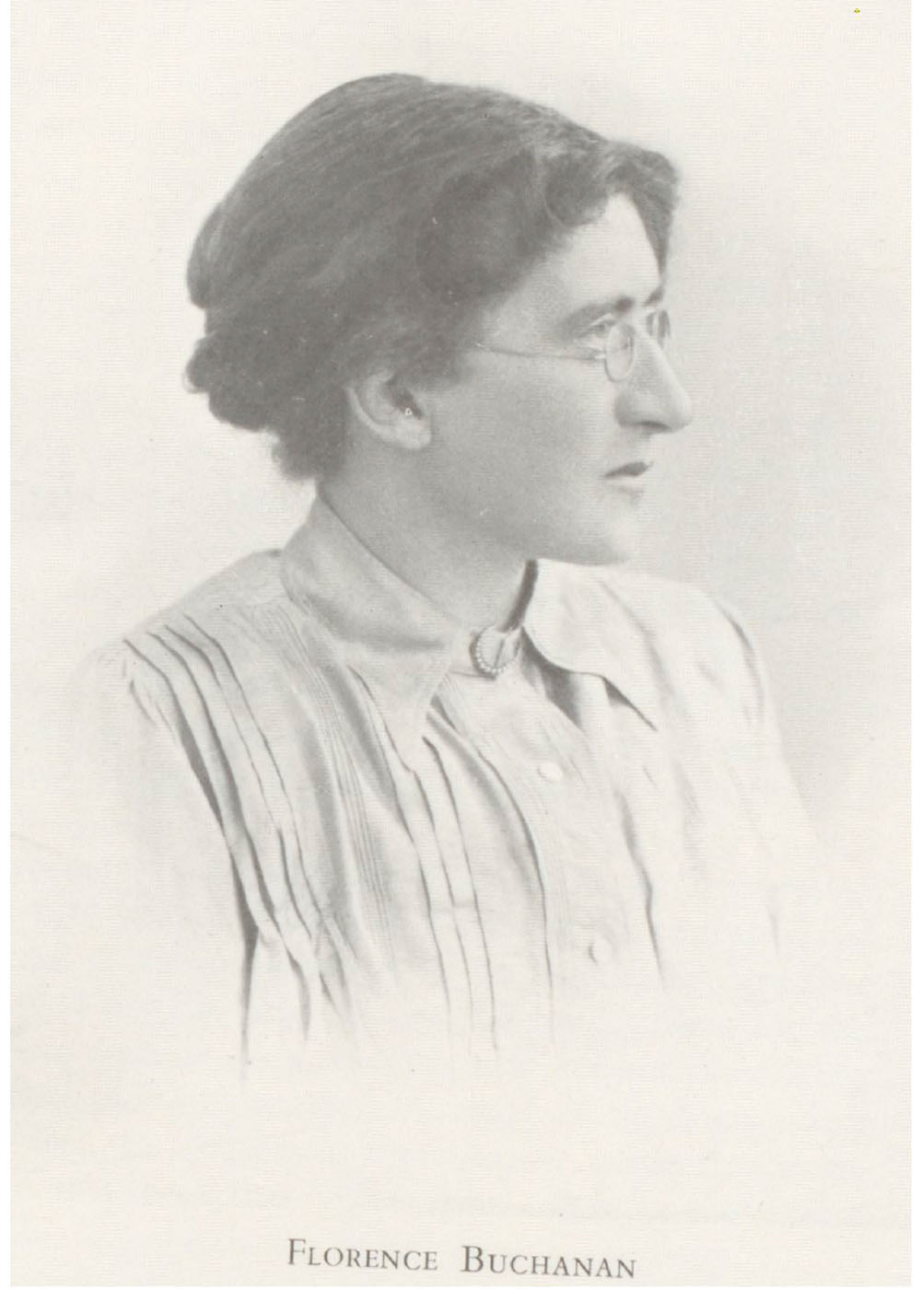
Dr Florence Buchanan, restored to view. Authenticated portrait from the Buchanan family memoir held by the National Library of Scotland, reproduced with permission and described by Clark et al. (2026).

The remaining presentations took Dr Buchanan's spirit of quantitative, integrative muscle physiology into contemporary science with a talented group of early career researchers (Figure 2). Dr Colleen S. Deane considered muscle health across the life course, emphasising skeletal muscle as both a locomotor and metabolic organ. Ageing is accompanied by an inexorable loss of muscle mass and strength, increasing frailty, falls, morbidity and mortality. Yet the mechanisms responsible for impaired adaptation to resistance exercise, nutrition and disuse remain incompletely defined. Multi‐omic approaches encompassing transcriptomics, proteomics and metabolomics now allow physiologists to move beyond phenotype alone, revealing molecular networks that coordinate adaptation across a multitude of biological layers. This approach is reflected in recent transcriptomic (Deane et al., 2021) and proteomic work comparing resistance exercise‐induced hypertrophy in young and older humans, highlighting avenues for therapeutic intervention. In this sense, contemporary muscle physiology continues Dr Buchanan's legacy: making otherwise hidden processes visible and measurable, taking full advantage of contemporary technologies.

**FIGURE 2:**
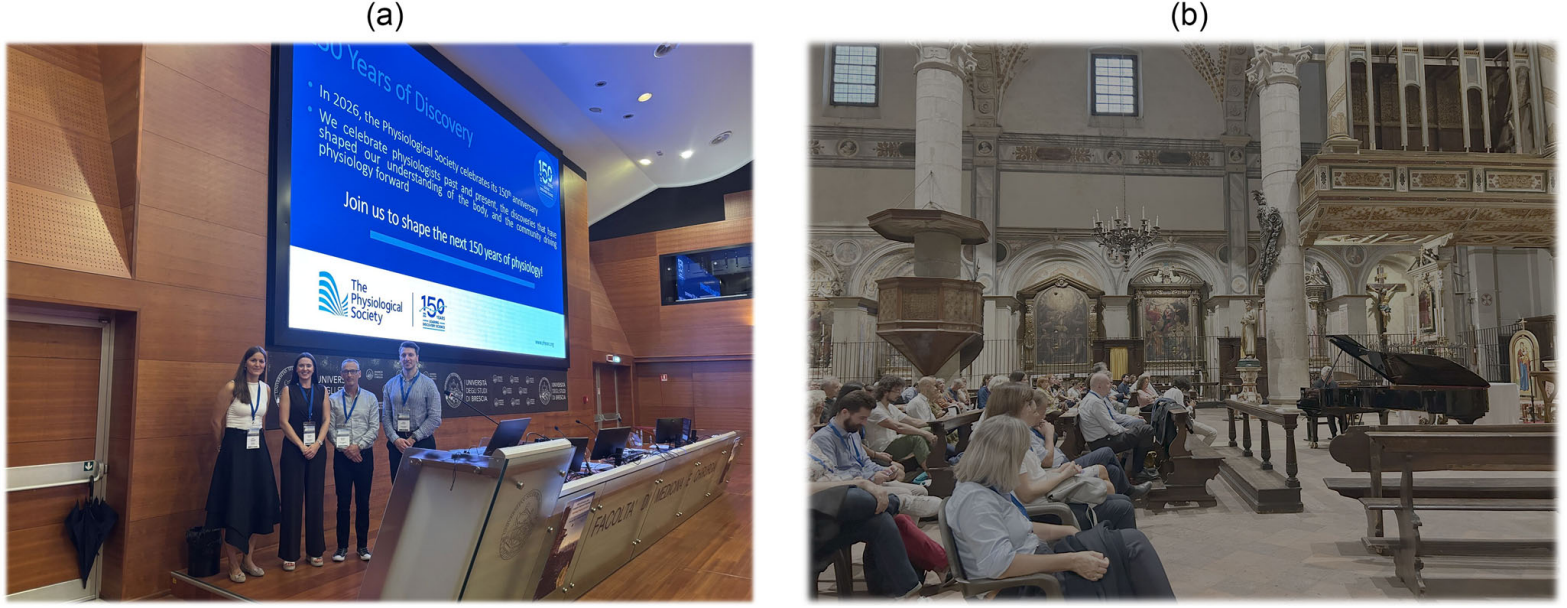
Speakers at the special event marking the 150th anniversary of The Physiological Society, ‘Building on the breakthroughs of Dr Florence Buchanan: advancing the frontiers of muscle physiology’, held during the Federation of European Physiological Societies and Italian Society of Physiology (FEPS–SIF) Brescia 2026 European Congress of Physiology. (a) Left to right: Dr Grith S. Højfeldt, Dr Colleen S. Deane, Professor Damian M. Bailey and Dr Jakob Škarabot. (b) The opening ceremony at the Chiesa di San Giuseppe, featuring the celebrated pianist Maestro Giorgio Costa, who performed selected works by Frédéric Chopin. Photographs reproduced courtesy of D.M.B. and The Physiological Society.

Dr Jakob Škarabot explored the neural–contractile nexus in healthy ageing, placing the motor unit at the centre of age‐related functional decline. Motor units translate neural activation into contractile output, and both sides of that transduction can deteriorate with age. Reduced motor unit discharge rates, altered estimates of persistent inward current contribution, slower contractile properties, reduced muscle volume and increased intramuscular fat collectively contribute to impaired force production. Importantly, these mechanisms appear muscle specific. In some muscles, weakness may be driven predominantly by neural changes (e.g. tibialis anterior); in others, morphology and tissue quality may dominate (e.g. quadriceps). Recent work showing age‐related differences in motor unit discharge‐rate modulation and estimates of persistent inward currents across contraction levels supports this view (Connelly et al., 2026). Together, these findings reinforce a central physiological principle: function is rarely explained at one level alone.

In the final presentation, Dr Grith Højfeldt addressed human skeletal muscle following injury, bringing the discussion from ageing to repair. Human skeletal muscle exhibits a remarkable regenerative capacity, but the cellular basis of durable recovery remains unresolved. Recent work from Højfeldt and colleagues has shown that nicotinamide and pyridoxine supplementation can enhance markers of muscle stem cell activity and muscle regeneration after high‐force eccentric muscle injury in humans (Hojfeldt et al. et al., 2026). Yet recovery of strength may precede complete extracellular matrix remodelling, perhaps helping explain recurrent injury. Once again, the theme is integrative, with repair not simply being contractile, but cellular, molecular, structural and mechanical.

Taken together, the symposium traced a line from Dr Buchanan's capillary electrometer to modern multi‐omics, motor unit physiology and regenerative biology. The tools have changed, but the physiological logic remains familiar: to reveal processes that are otherwise hidden, measure them carefully, and understand how they shape human function. In this respect, each speaker extended Dr Buchanan's legacy, moving from synaptic delay to molecular adaptation, neural drive and tissue remodelling.

The connection between Dr Buchanan's work and contemporary muscle physiology is more than historical coincidence. A 150th anniversary invites not only commemoration, but reflection on how the discipline's history should shape its future. Dr Buchanan's legacy rests partly on her three ‘fearless firsts’ and on the wider need to mind and mend the visibility gap for women in physiology (Bailey, 2023). It also lies in how she practised physiology: with precision, independence, technical imagination, interpretive restraint and due recognition of collaboration. Her work reminds us that progress depends on clearer measurement and a community willing to recognise contributions that earlier generations too often overlooked.

As The Physiological Society enters its next 150 years, Dr Buchanan offers a powerful lens through which to consider both science and culture. Her work helped define the measurable physiology of nerve and muscle, while her career exposed the exclusions that shaped the institutions of her time. Set against the backdrop of Brescia, FEPS–SIF 2026 and Italy's rich tradition of integrative physiology, her story reminds us that physiology advances through careful measurement, shared lineages, international exchange and communities willing to carry ideas forward. To build on her breakthroughs is therefore not only to honour an historical pioneer, but to continue her work: measuring carefully, seeing more clearly despite the limitations imposed by her near blindness, and ensuring that physiology's future is shaped by the full breadth of those capable of advancing it.

## AUTHOR CONTRIBUTIONS

Damian M. Bailey conceived the idea and wrote the first draft of the manuscript. Damian M. Bailey, Colleen S. Deane, Jakob Škarabot, Grith Højfeldt and Guido Ferretti approved the final version submitted for publication and agree to be accountable for all aspects of the work in ensuring that questions related to the accuracy or integrity of any part of the work are appropriately investigated and resolved. All persons designated as authors qualify for authorship, and all those who qualify for authorship are listed.

## CONFLICT OF INTEREST

Damian M. Bailey is Editor‐in‐Chief of *Experimental Physiology*. Colleen S. Deane is a Senior Editor of *Experimental Physiology*.

## GENERATIVE AI STATEMENT

The authors confirm that no artificial intelligence tools, including large language models, were used in the drafting, revision or intellectual development of this manuscript. All content was conceived, written, reviewed and approved solely by the authors.
